# Radioprotective effects of cimetidine on rats irradiated by long-term, low-dose-rate neutrons and ^60^Co γ-rays

**DOI:** 10.1186/s40779-017-0116-7

**Published:** 2017-02-27

**Authors:** Ding-Wen Jiang, Qing-Rong Wang, Xian-Rong Shen, Ying He, Tian-Tian Qian, Qiong Liu, Deng-Yong Hou, Yu-Ming Liu, Wei Chen, Xin Ren, Ke-Xian Li

**Affiliations:** The PLA Key Laboratory of Biological Effect and Medical Protection on Naval Vessel Special Environment, Naval Medical Research Institute, Xiangyin Road 880, Shanghai, 200433 China

**Keywords:** Cimetidine, Radioprotection, Antioxidation, Immunomodulation, Micronuclei

## Abstract

**Background:**

Cimetidine, an antagonist of histamine type II receptors, has shown protective effects against γ-rays or neutrons. However, there have been no reports on the effects of cimetidine against neutrons combined with γ-rays. This study was carried out to evaluate the protective effects of cimetidine on rats exposed to long-term, low-dose-rate neutron and γ-ray combined irradiation (n-γ LDR).

**Methods:**

Fifty male Sprague-Dawley (SD) rats were randomly divided into 5 groups: the normal control group, radiation model group, 20 mg/(kg · d) cimetidine group, 80 mg/(kg · d) cimetidine group and 160 mg/(kg · d) cimetidine group (10 rats per group). Except for the normal control group, 40 rats were simultaneously exposed to fission neutrons (^252^Cf, 0.085 mGy/h) for 22 h every day and γ-rays (^60^Co, 0.097 Gy/h) for 1.03 h once every three days, and the cimetidine groups were administered intragastrically with cimetidine at doses of 20, 80 and 160 mg/kg each day. Peripheral blood WBC of the rats was counted the day following exposure to γ-rays. The rats were anesthetized and sacrificed on the day following exposure to ^252^Cf for 28 days. The spleen, thymus, testicle, liver and intestinal tract indexes were evaluated. The DNA content of bone marrow cells and concanavalin A (ConA)-induced lymphocyte proliferation were measured. The frequency of micronuclei in polychromatic erythrocytes (fMNPCEs), superoxide dismutase (SOD), malondialdehyde (MDA), and glutathione peroxidase (GSH-Px) in the serum and liver tissues were detected.

**Results:**

The peripheral blood WBC in the cimetidine groups was increased significantly on the 8th day and the 26th day compared with those in the radiation model group. The spleen, thymus and testicle indexes of the cimetidine groups were higher than those of the radiation model group. The DNA content of bone marrow cells and lymphocyte proliferation in the cimetidine groups were increased significantly, and fMNPCE was reduced 1.41-1.77 fold in cimetidine treated groups. The activities of SOD and GSH-Px in the cimetidine groups were increased significantly, and the content of MDA in the cimetidine groups was decreased significantly.

**Conclusions:**

The results suggested that cimetidine alleviated damage induced by long-term, low-dose-rate neutron and γ combined irradiation *via* antioxidation and immunomodulation. Cimetidine might be useful as a potent radioprotector for radiotherapy patients as well as for occupational exposure workers.

## Background

Most of the data on radioprotection have been derived from studies using low linear energy transfer (LET) X- and γ-radiation. Exposure to high LET radiation can also be expected during long-term space flights, occupational exposure in nuclear plants, and tumor radiotherapy. It was demonstrated that neutrons and other high LET radiation induce more severe biological effects than low LET radiation for the same absorbed dose [1, 2]. Thus, protection against high LET radiation should be given more attention. It was observed that cysteine with a dose reduction factor (DRF) of 1.7 for γ-rays was shown to have a DRF of only 1.1 in mice against neutrons [3]. From the discovery of the radio-protective effect of cysteine, increasingly more compounds have been used in an attempt to provide partial protection against radiation injury. However, thus far, sulfhydryl compounds remain the main radioprotectors, such as mercapto-ethylamine (MEA), aminethylisothiouronium (AET) and amifostine (WR-2721) [4]. However, the side effects of these radioprotectors have limited their clinical application [5]. Therefore, the development of new drugs that are less toxic and effective even for high LET exposure is desired for the protection of medical and occupational exposure to radiation.

Cimetidine, an antagonist of histamine type II receptors that is usually used for peptic ulcer treatment, has shown protective effects against radiation, not only high LET neutrons but also low LET γ or X-rays. Cimetidine was shown to be effective against the clastogenic effects of γ-rays [6, 7] and X-rays [8] in vitro. It was also demonstrated to be protective against γ-rays and neutrons in vivo [7, 9–12]. Cimetidine was shown to inhibit the activity of suppressor T cells [13] and was used in radioprotection to help the recovery of the lymphohematopoietic system effectively [10]. H_2_-receptor antagonists have potential oxygen-radical-scavenging properties. It was reported that cimetidine could scavenge OH with a very high rate constant [14]. Mice treated with cimetidine could tolerate biomembrane damage provoked by sublethal γ-radiation. This supports the hypothesis that cimetidine may afford efficient protection against ionizing radiation or diseases that are characterized by in vivo free radical-mediated oxidative stress mechanisms [15]. It was shown that cimetidine, ranitidine, and famotidine produced a DRF of 1.5 to 2 against γ-ray-induced micronuclei in mouse bone marrow erythrocytes [9, 16]. Cimetidine, compared with famotidine, was found to be more protective against mortality induced by radiation in mice [12].

In this study, SD rats were exposed to long-term, low-dose-rate neutron and γ combined irradiation (n-γ LDR) with or without cimetidine supplementation, and the protective effects of cimetidine on rats exposed to n-γ LDR were reported.

## Methods

### Materials

Cimetidine was purchased from Tianjin Smith Kline &French Laboratories Ltd (Tianjin, China). Superoxide dismutase (SOD), malondialdehyde (MDA), and glutathione peroxidase (GSH-Px) kits were purchased from the Nanjing Jiancheng Bioengineering Institute.

### Animals and treatments

Five-week-old male SD rats were purchased from Sino-British SIPPR/BK Lab. Animal Co. Ltd. The rats were maintained under conditions of standard lighting (12:12 h light–dark cycle), temperature (20–22 °C) with freely available food and water. The animals were randomly divided into 5 groups (*n* = 10): normal control group, radiation model group, 20 mg/(kg · d) cimetidine group, 80 mg/(kg · d) cimetidine group and 160 mg/(kg.d) cimetidine group. Cimetidine was administered by intragastric infusion with the volume of 10 ml/kg each day. The Institutional Animal Care and Use Committees of the Naval Medical Research Institute approved all procedures for animal care and treatment.

### Animal irradiation

From the first day of cimetidine administration, animals, except those in the normal control group, were whole-body exposed to fission neutrons (^252^Cf, 0.085 mGy/h, the Radiation Center of the Naval Medical Research Institute) 22 h every day for 28 days, and γ-rays (^60^Co, 0.097 Gy/h, the Radiation Center of the Naval Medical Research Institute) 1.03 h once every three days. The cumulative doses were 52.36 mGy of neutron and 1.0 Gy of γ-rays. The radiation method and doses are shown in Table 1.

**Table 1:** Accumulated dose of n-γ LDR on the day of vein blood collection

Radiation	8th day	17th day	26th day	29th day
Neutron (mGy)	13.09	29.92	46.75	52.36
γ-rays (Gy)	0.3	0.6	0.9	1.0

The 8th, 17th, 26th and 29th day indicates the time points of vein blood collection; at these time points, the rats were actually exposed to n-γ LDR for 7, 16, 25 and 28 days

### Peripheral blood white blood cell (WBC) count

Whole blood was collected from the rat tail into EDTA-coated tubes on the 3rd day before radiation and on the 8th, 17th and 26th days after radiation, and the absorbed doses of neutrons and γ-rays on these days are shown in Table 1. Peripheral blood WBC counts were obtained using a veterinary hematology analyzer (Nihon Kohden Corporation, Japan) according to the operator’s manual.

### Organ weight index detection

Animals were anesthetized and sacrificed on the next day after exposure to ^252^Cf for 28 days. The spleen, thymus, testicle, liver, and intestinal tract were separated, and organ indexes were determined using the following equation: Organ index = organ weight/body weight.

### Bone marrow DNA content detection

The next day after finished all the irradiation, the right femur of each animal was removed and cleaned of adherent tissues. The bone marrow was flushed into 5 ml of a 5-mmol/L CaCl_2_ solution and was incubated at 4 °C for 30 min. Cell suspensions were centrifuged at 2,500 r/min for 15 min, and the pellet was resuspended in 5 ml 0.2 mol/L HClO_4_. The suspension was incubated at 90 °C for 15 min and was filtered through a 0.22-μm membrane. The absorbance at 260 nm was detected using a 759 UV spectrophotometer (Shanghai APL instrument Co., Ltd.).

### Detection of SOD, MDA, and GSH-Px in serum and hepatic tissue

The next day after finished all the irradiation, blood was collected from the inferior vena cava into clean centrifuge tubes. Serum was prepared by centrifugation at 3,000 × *g* for 15 min. Next, 100 mg of hepatic tissue filled with 0.9 ml of normal saline was homogenized with a pulping machine, and the supernatant was prepared by centrifugation at 3,000 × *g* for 15 min. SOD, MDA, and GSH-Px in serum and hepatic tissue supernatants were determined using detecting kits from Nanjing Jiancheng Bioengineering Institute according to the manufacturer’s instructions.

### Concanavalin A (ConA)-induced splenocyte proliferation assay

The next day after finished all the irradiation, splenic lymphocytes were prepared as previously described [17]. One milliliter of spleen cell suspension (3 × 10^6^ cells/ml) was added into 24-well plates and cultured with 75 μl of 100 μg/ml ConA solution. The plates were incubated at 37 °C with 5% CO_2_ for 68 h, and then 0.7 ml of supernatant in each well was discarded and 0.7 ml of RPMI 1640 medium was added. Next, 50 μl of fresh prepared MTT solution (5 mg/ml, dissolved in PBS, pH 7.2) was added into each well and incubated under the same conditions for 4 h. Finally, 1 ml of acid-isopropanol solution (1 mol/L HCl/isopropanol was 4/96(*v/v*)) was added into each well and mixed thoroughly until the purple crystals were fully dissolved. The absorbance at 570 nm was determined.

### Detection of the frequency of micronuclei in polychromatic erythrocyte (fMNPCE) in the bone marrow

The next day after finished all the irradiation, bone marrow samples were collected from the left femur of each animal. The bone marrow was flushed into 1.5-ml tube used by 400 μl of fetal bovine serum (FBS), and then 200 μl of suspension was fixed by adding 5.0 ml of 1% glutaraldehyde solution containing 30 μl/ml SDS and 0.05 mol/L Sorensen. The fixed cells were incubated for 5 min and then were centrifuged at 300 × *g* for 5 min. The cell pellets were suspended in 1.0 ml of acridine orange staining solution and staining for 30 min at 37 °C. The stained cells were analyzed using the Accuri C6 flow cytometer (BD Biosciences, NJ, USA). At least 10,000 cells/sample were collected, and the fMNPCE was analyzed.

### Statistical analysis

Statistical analysis was performed by one-way analysis of variance (AVOVA) followed by LSD *t*-test using SPSS (version 16.0). All of the values were expressed as mean as the means ± standard deviation (SD). *P* values <0.05 were considered to be statistically significant.

## Results

### Protection effect of cimetidine on peripheral blood WBC of irradiated rats

The results are shown in Table 2. Peripheral blood WBC in all groups was not significantly different on the 3rd day before radiation (*P* > 0.05). WBC in the irradiation groups was decreased significantly on the 8th, 17th and 26th days, respectively (*P* < 0.01). On the 8th day, WBC in the cimetidine groups was increased significantly compared with that in the radiation model group (*P* < 0.01 in the 20 mg/(kg · d) group, *P* < 0.05 in the 80 mg/(kg · d) group). On the 26th day, WBC in the 20 mg/(kg · d) and 80 mg/(kg · d) cimetidine groups was increased significantly compared with that in the radiation model group (*P* < 0.05). These results suggested that cimetidine has a promotion effect on peripheral blood WBC of n-γ LDR rats.

**Table 2:** Effect of cimetidine on peripheral blood WBC counts in n-γ LDR rats (×10^9^/L, *x* ± *s*, *n* = 10)

Group	3rd day before IR	8th day	17th day	26th day
Normal control group	10.9 ± 1.6	11.6 ± 1.2	10.7 ± 1.2	10.0 ± 2.6
Radiation model group	10.8 ± 2.2	7.7 ± 1.0^(1)^	8.2 ± 2.3^(1)^	6.3 ± 0.7^(1)^
20 mg/(kg · d) cimetidine group	10.7 ± 1.5	11.3 ± 2.1^(3)^	8.6 ± 1.3^(1)^	7.2 ± 1.2^(1)(2)(4)^
80 mg/(kg · d) cimetidine group	10.6 ± 1.7	9.0 ± 1.6^(1)(2)^	7.9 ± 1.1^(1)^	7.2 ± 1.0^(1)(2)(4)^
160 mg/(kg · d) cimetidine group	10.5 ± 1.5	9.0 ± 1.9^(1)^	8.4 ± 1.2^(1)^	5.8 ± 1.2^(1)^

^(1)^ *P* < 0.01 compared with the normal control group; ^(2)^ *P* < 0.05, ^(3)^ *P* < 0.01 compared with the radiation model group; ^(4)^ *P* < 0.05 compared with the 160 mg/(kg · d) cimetidine group. The 8th, 17th and 26th day were the days that the rats were exposed to n-γ LDR for 7, 16 and 25 days, respectively

### Protection effect of cimetidine on the organ indexes of irradiated rats

The results are shown in Table 3. The spleen indexes, thymus indexes, and testicle indexes of the radiation model group were decreased significantly compared with those of the normal control group (*P* < 0.05 or *P* < 0.01), and the liver indexes and intestinal tract indexes showed no significant difference (*P* > 0.05). The spleen indexes of the cimetidine groups were increased compared with those of the radiation model group (*P* < 0.01 in the 20 mg/(kg · d) cimetidine group and *P* < 0.05 in the 160 mg/(kg · d) cimetidine group). The thymus indexes of the 20 mg/(kg · d) cimetidine group were increased compared with those of the radiation model group (*P* < 0.05). The testicle indexes of the 160 mg/(kg · d) cimetidine group were increased compared with those of the radiation model group (*P* < 0.05). The liver indexes and intestinal tract indexes in all groups showed no significant difference (*P* > 0.05). These results suggested that n-γ LDR induced the damage effects on the spleen, thymus and testicle, and cimetidine alleviated the damage effects of the three organs in irradiated rats.

**Table 3:** Effect of cimetidine on the organ indexes of n-γ LDR rats (mg/g, *x* ± *s*, *n* = 10)

Group	Spleen index	Thymus index	Testicle index	Liver index	Intestinal tract index
Normal control group	2.16 ± 0.21	1.75 ± 0.14	8.20 ± 0.83	33.75 ± 4.01	0.38 ± 0.03
Radiation model group	1.74 ± 0.22^(2)^	1.41 ± 0.26^(1)^	7.44 ± 0.46^(1)^	32.81 ± 1.63	0.37 ± 0.03
20 mg/(kg · d) cimetidine group	2.02 ± 0.23^(4)^	1.69 ± 0.29^(3)^	7.88 ± 0.58	32.03 ± 1.24	0.38 ± 0.02
80 mg/(kg · d) cimetidine group	1.89 ± 0.21^(1)^	1.64 ± 0.31	7.60 ± 0.61	31.63 ± 2.15	0.38 ± 0.02
160 mg/(kg · d) cimetidine group	1.91 ± 0.14^(2)(3)^	1.63 ± 0.29	7.94 ± 0.49^(3)^	34.34 ± 3.25	0.39 ± 0.03

^(1)^ *P* < 0.05, ^(2)^ *P* < 0.01 compared with the normal control group; ^(3)^ *P* < 0.05, ^(4)^ *P* < 0.01 compared with the radiation model group

### Effect of cimetidine on the DNA content of bone marrow cells

**Fig. 1:**
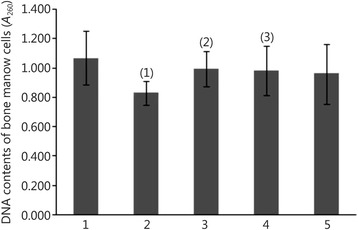
Effect of cimetidine on the DNA content of bone marrow cells in irradiated rats. 1. Normal control group; 2. Radiation model group; 3. 20 mg/(kg · d) cimetidine group; 4. 80 mg/(kg · d) cimetidine group; 5. 160 mg/(kg · d) cimetidine group. (1) *P* < 0.01 compared with the normal control group; (2) *P* < 0.01, (3) *P* < 0.05 compared with the radiation model group

The results are shown in Fig. 1. The DNA content of bone marrow cells in the radiation model group was decreased significantly compared with that in the normal control group (*P* < 0.01), and the DNA content in the cimetidine groups was increased compared with that in the radiation model group (*P* < 0.01 in the 20 mg/(kg · d) cimetidine group and *P* < 0.05 in the 80 mg/(kg · d) cimetidine group). These results suggested that bone marrow cells are damaged by n-γ LDR, and cimetidine has a protective effect on the DNA of bone marrow cells in irradiated rats.

### Effect of cimetidine on ConA-induced splenocyte proliferation

**Fig. 2:**
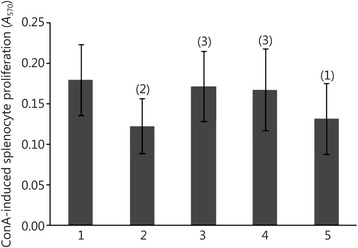
Effect of cimetidine on ConA-induced splenocyte proliferation in rats. 1. Normal control group; 2. Radiation model group; 3. 20 mg/(kg · d) cimetidine group; 4. 80 mg/(kg · d) cimetidine group; 5. 160 mg/(kg · d) cimetidine group. (1) *P* < 0.05, (2) *P* < 0.01 compared with the normal control group; (3) *P* < 0.05 compared with the radiation model group

Figure 2 shows that ConA-induced splenocyte proliferation in the radiation model group was decreased significantly compared with that in the normal control group (*P* < 0.01), while in the 20 mg/(kg · d) or 80 mg/(kg · d) cimetidine group was increased significantly compared with that in the radiation model group (*P* < 0.05). The results suggested that n-γ LDR have an injured effect on the splenocytes of the irradiated rats, and cimetidine ameliorated the damage, thus improving the splenocyte proliferation in irradiated rats.

### Effect of cimetidine on SOD, GSH-Px and MDA in serum and hepatic tissue

Table 4 shows that the SOD activity in serum and hepatic tissue of the radiation model group was decreased significantly compared with that of the normal control group (*P* < 0.01), and the SOD activity in the 20 mg/(kg · d) and 160 mg/(kg · d) cimetidine group of serum and in the 20 mg/(kg · d) cimetidine group of hepatic tissue were increased significantly compared with that in the radiation model group (*P* < 0.05 or *P* < 0.01). Table 5 shows that the GSH-Px activity in the serum of the radiation model group was decreased significantly compared with that of the normal control group (*P* < 0.05), and the GSH-Px activity in serum of the 20 mg/(kg · d) and 80 mg/(kg · d) cimetidine group was increased significantly compared with that of the radiation model group (*P* < 0.05). The GSH-Px activity in hepatic tissue showed no significant difference of all groups (*P* > 0.05). These results suggested that n-γ LDR decreased the antioxidation activities of rats, and cimetidine had the effect of stimulating the activities of antioxidation in n-γ LDR rats. Table 6 shows that the MDA in serum and hepatic tissue of the radiation model group was increased significantly compared with that of the normal control group (*P* < 0.05 or *P* < 0.01). The content of MDA in the 20 mg/(kg · d) and 160 mg/(kg · d) cimetidine group in serum and in all cimetidine groups in hepatic tissue was decreased remarkably compared with that in the radiation model group (*P* < 0.05 or *P* < 0.01). These results suggested that cimetidine had the effect of stimulating serum and hepatic tissue MDA elimination in n-γ LDR rats.

**Table 4:** Effect of cimetidine on SOD activity in serum and hepatic tissues of n-γ LDR rats (*x* ± *s*, *n* = 10)

Group	SOD in serum (U/ml)	SOD in hepatic tissue (U/mg protein)
Normal control group	20.22 ± 0.94	174.01 ± 20.84
Radiation model group	15.04 ± 1.60^(2)^	142.99 ± 4.03^(2)^
20 mg/(kg · d) cimetidine group	19.75 ± 5.39^(4)^	184.25 ± 7.59^(4)^
80 mg/(kg · d) cimetidine group	16.26 ± 3.60^(1)^	137.87 ± 26.99^(2)(5)^
160 mg/(kg · d) cimetidine group	17.47 ± 2.13^(2)(3)^	141.59 ± 11.04^(2)(5)^

^(1)^ *P* < 0.05, ^(2)^ *P* < 0.01 compared with the normal control group; ^(3)^ *P* < 0.05, ^(4)^ *P* < 0.01 compared with the radiation model group; ^(5)^ *P* < 0.05 compared with the 20 mg/(kg · d) cimetidine group

**Table 5:** Effect of cimetidine on GSH-Px activity in serum and hepatic tissues of n-γ LDR rats (*x* ± *s, n* = 10)

Group	GSH-Px in serum (U/ml)	GSH-Px in hepatic tissue (U/mg protein)
Normal control group	1533.07 ± 222.34	230.67 ± 37.99
Radiation model group	1307.20 ± 178.43^(1)^	215.73 ± 21.05
20 mg/(kg · d) cimetidine group	1455.85 ± 103.16^(2)^	208.6 ± 12.90
80 mg/(kg · d) cimetidine group	1466.47 ± 173.53^(2)^	222.94 ± 29.92
160 mg/(kg · d) cimetidine group	1423.29 ± 191.34	210.05 ± 32.30

^(1)^ *P* < 0.05 compared with the normal control group; ^(2)^ *P* < 0.05 compared with the radiation model group

**Table 6:** Effect of cimetidine on MDA in serum and hepatic tissues of n-γ LDR rats (*x* ± *s, n* = 10)

Group	MDA in serum (nmol/ml)	MDA in hepatic tissue (nmol/mg protein)
Normal control group	3.67 ± 0.431	4.18 ± 0.60
Radiation model group	4.02 ± 0.37^(2)^	4.89 ± 0.28^(1)^
20 mg/(kg · d) cimetidine group	3.70 ± 0.36^(3)^	4.34 ± 0.29^(4)^
80 mg/(kg · d) cimetidine group	3.83 ± 0.40	4.35 ± 0.53^(3)^
160 mg/(kg · d) cimetidine group	3.50 ± 0.38^(4)^	4.27 ± 0.30^(4)^

^(1)^ *P* < 0.05, ^(2)^ *P* < 0.01compared with the normal control group; ^(3)^ *P* < 0.05, ^(4)^ *P* < 0.01 compared with the radiation model group

### Effect of cimetidine on the fMNPCE in rat bone marrow erythrocytes

**Fig. 3:**
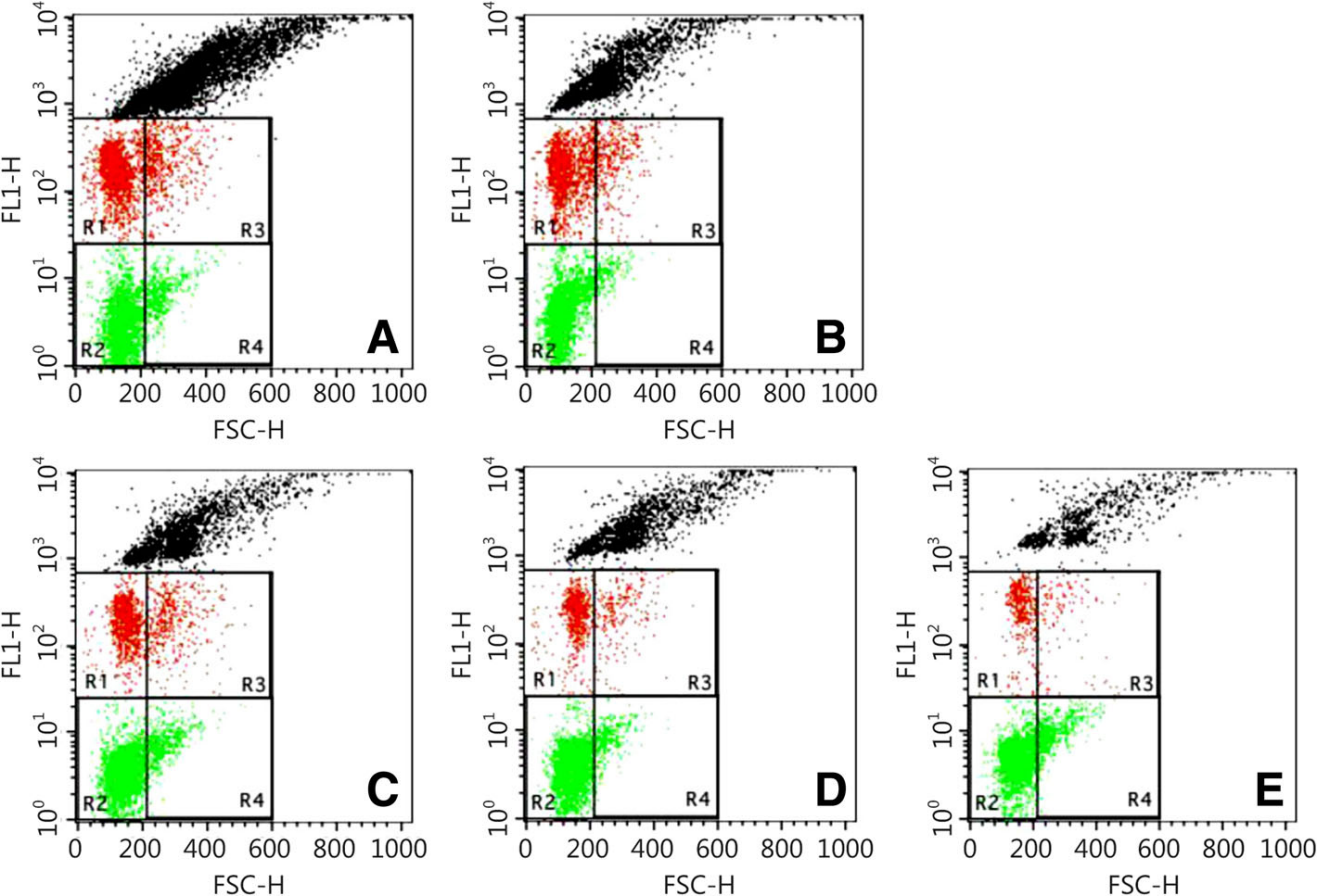
FACS of rats on the frequencies of micronucleated polychromatic erythrocytes. **a** Normal control group; **b** Radiation model group; **c** 20 mg/(kg · d) cimetidine group; **d** 80 mg/(kg · d) cimetidine group; **e** 160 mg/(kg · d) cimetidine group

The results are shown in Fig. 3 and Table 7. The fMNPCE in the radiation group was increased significantly compared with that in the normal control group (*P* < 0.01), and the fMNPCE values in the three cimetidine-treated groups were decreased significantly compared with that in the radiation model group (*P* < 0.01). The DRF values of MN in the 20 mg/(kg · d), 80 mg/(kg · d) and 160 mg/(kg · d) cimetidine group were 1.41, 1.77 and 1.59, respectively. These results suggested that cimetidine reduced the number of micronuclei in bone marrow polychromatic erythrocytes of irradiated rats, and protected against DNA damage induced by radiation.

**Table 7:** Effect of cimetidine on the fMNPCE in bone marrow erythrocytes of n-γ LDR rats (*x* ± *s, n* = 10)

Group	fMNPCE (%)	DRF
Normal control group	0.150 ± 0.0216	—
Radiation model group	0.410 ± 0.0267^(1)^	—
20 mg/(kg · d) cimetidine	0.290 ± 0.0213^(1)(2)^	1.41
80 mg/(kg · d) cimetidine	0.231 ± 0.0325^(1)(2)^	1.77
160 mg/(kg · d) cimetidine	0.258 ± 0.0268^(1)(2)^	1.59

^(1)^ *P* < 0.01 compared with the normal control group; ^(2)^ *P* < 0.01 compared with the radiation model group; “—”. No data

## Discussion

Cimetidine can protect cells against the clastogenic effects of γ-rays or low-dose fast neutrons [6–9, 11]. However, there have been no reports on the effects of low-dose-neutron combined γ-rays. In our study, the rats were treated with whole-body n-γ LDR and administered cimetidine at 20, 80 and 160 mg/(kg · d) by intragastric infusion. The results showed that the fMNPCE of all cimetidine-treated rats was reduced remarkably, with a DRF of 1.41, 1.77 and 1.59, respectively. This suggested that cimetidine can protect bone marrow polychromatic erythrocytes against the clastogenic effects of n-γ LDR.

Ionizing radiation (IR) injury has been attributed to reactive oxygen species (ROS)-mediated lipid peroxidation, which can be estimated by measuring the concentration of MDA. In the present study, cimetidine treatment rats showed significantly reduced MDA levels in the serum and liver, suggesting that cimetidine may diminish n-γ LDR injury by decreasing ROS levels and reducing oxidative damage.

Biological systems protect themselves against the damaging effects of activated species by several means, including free radical scavengers and enzymes such as SOD and the GSH-Px system. It was reported that antioxidants could reduce IR injury. H_2_-receptor antagonists are scavengers of hydroxyl radicals with a very high rate constant [8, 18]. They can stimulate SOD activity and decrease the MDA concentration in the blood of patients with peptic ulcer disease [19]. Mice treated with cimetidine could tolerate biomembrane damage provoked by sublethal γ-radiation [15]. The results in this study showed that SOD and GSH-Px activities in the serum were decreased significantly in n-γ LDR rats. The activity of SOD and GSH-Px was increased by treatment with cimetidine. Treatment with cimetidine possibly inhibited the accumulation of n-γ LDR-induced free radicals, decreased the oxidative stress and protected the antioxidant enzymes of rats as revealed by the enhanced activities of SOD and GSH-Px in this study. The results supported the hypothesis that cimetidine might afford an efficient protection against IR or diseases that were characterized by in vivo free radical-mediated oxidative stress mechanisms [15].

Radiation-induced lymphohematopoietic syndrome is characterized by a depression in the peripheral blood levels of white and red blood cells and platelets, as well as the loss of weight and decrease in the size of lymphatic tissues such as the spleen and thymus gland. In this study, cimetidine was shown to have an effect of increasing the number of WBC and DNA content of bone marrow cells. These results suggested that cimetidine may play an important role in protecting the hematopoietic system against n-γ LDR in vivo. This study also showed that the spleen indexes, thymus indexes and splenocyte proliferation of cimetidine-treated rats were increased significantly. It also suggested that cimetidine can improve the immune function of irradiated rats. Recent studies have shown that cimetidine could modulate immune responses. Cimetidine might act as a non-specific stimulant of cell-mediated immunity and as an immunomodulator [20], and enhances antigen-specific IgE and Th2 cytokine production [21]. Cimetidine augments Th1/Th2 dual polarized immune responses to recombinant HBV antigens [22]. It has also been revealed that a group of T cells with histamine receptors might have a suppressive effect. Therefore, an antagonist of histamine type II receptors might play a role in the immune system and inhibit the function of suppressor T cells, mainly by increasing the CD4^+^ cells of peripheral blood T lymphocytes [13, 23]. The administration of cimetidine prior to irradiation leads to the inhibition of T suppressor cells and an increase in the proliferation of CD4^+^ lymphocytes, causing the production of glutathione reductase and catalase enzymes, which prevent DNA damage and eventually reduce the clastogenic effect of radiation [13, 24]. Thus, cimetidine might, *via* the regulation T lymphocytes, protect rats against immune and oxidative damage by n-γ LDR. These results suggested that cimetidine may be a useful candidate radioprotector for low-dose occupational radiation.

## Conclusion

The effect of cimetidine on rats exposed to long-term n-γ LDR was evaluated in this study. The results showed that cimetidine increased the peripheral blood WBC, spleen, thymus and testicle indexes, DNA content of marrow cells, lymphocyte proliferation, and activities of SOD and GSH-Px in n-γ LDR rats significantly but reduced the frequency of micronuclei in polychromatic erythrocytes (1.41–1.77 fold) and effectively decreased the content of MDA in n-γ LDR rats. The results suggested that cimetidine can alleviate damage induced by n-γ LDR *via* antioxidation and immunomodulation. Cimetidine might be useful as a potent radioprotector for radiotherapy patients as well as occupational exposure workers.

## Abbreviations

AET: Aminoethylisothiouronium
ConA: Concanavalin A
DRF: Dose reduction factor
FBS: Fetal bovine serum
fMNPCE: Frequency of micronuclei in polychromatic erythrocytes
GSH-Px: Glutathione peroxidase
IR: Ionizing radiation
LET: Low linear energy transfer
MDA: Malondialdehyde
MEA: Mercapto-ethylamine
n-γ LDR: Low-dose-rate neutron and γ-ray combined irradiation
ROS: reactive oxygen species
SD: Sprague-Dawley
SOD: Superoxide dismutase
WBC: White blood cell
